# Nephroprotective Effects of Saponins from Leaves of *Panax quinquefolius* against Cisplatin-Induced Acute Kidney Injury

**DOI:** 10.3390/ijms18071407

**Published:** 2017-07-13

**Authors:** Zhi-Na Ma, Yan-Zi Li, Wei Li, Xiao-Tong Yan, Ge Yang, Jing Zhang, Li-Chun Zhao, Li-Min Yang

**Affiliations:** 1 College of Chinese Medicinal Materials, Jilin Agricultural University, Changchun 130118, China; mazhina9029@163.com (Z.-N.M.); liyanzi0401@126.com (Y.-Z.L.); yanxiaotong0707@126.com (X.-T.Y.); yangge1900@163.com (G.Y.); zhjing@163.com (J.Z.); 2 College of Pharmacy, Guangxi University of Chinese Medicine, Nanning 530001, China; hyzlc@126.com

**Keywords:** ginsenosides, the leaves of *Panax quinquefolium*, cisplatin-induced AKI, anti-inflammation, anti-apoptosis

## Abstract

Although cisplatin is an anticancer drug that has activity against malignant tumor, it often causes nephrotoxicity. Previous reports have confirmed that the saponins from the leaves of *P. quinquefolium* (PQS) exerted many pharmacological activities. However, the renoprotective effects of PQS were still unknown. The purpose of the present research was to discuss renoprotective effect of PQS in a mouse model of cisplatin-induced acute kidney injury (AKI). The levels of blood urea nitrogen (BUN) and serum creatinine (CRE) were evidently increased in cisplatin-intoxicated mice, which were reversed by PQS. Renal oxidative stress, evidenced by increased malondialdehyde (MDA) level and decline of glutathione (GSH) and superoxide dismutase (SOD) activities, was significantly alleviated by PQS pretreatment. The suppression of inflammatory response by PQS was realized through the decrease the mRNA expression levels of tumor necrosis factor-α (*TNF-α*) and interleukin-1β (*IL-1β*) in kidney tissues, which were measured by quantitative real-time polymerase chain reaction (qRT-PCR). Simultaneously, the overexpression of cytochrome P450 E1 (CYP2E1) and heme oxygenase-1 (HO-1) were attenuated by PQS. Furthermore, the effects of Western blotting demonstrated that PQS administration significantly suppressed the protein expression levels of nicotinamide adenine dinucleotide phosphate oxidase type 4 (Nox4), cleaved Caspase-3, cleaved Caspase-9, Bax, nuclear factor-κB (NF-κB), cyclooxygenase-2 (COX-2), and inducible nitric oxide synthase (iNOS), suggesting the inhibition of apoptosis and inflammation response. Overall, PQS may possess protective effects in cisplatin-induced AKI through suppression of oxidative stress, inflammation and apoptosis.

## 1. Introduction

Cisplatin, a potent anti-cancer drug, is widely used and highly effective against multiple types of tumors, involving breast, ovarian, head and neck, and lung [1]. However, its side effects restrict its clinical use. Clinical trials have reported that the side effects of cisplatin included nephrotoxicity, electrolyte disturbance, myelotoxicity, hemolytic anemia, neurotoxicity and ototoxicity [2]. Among its side effects, the main dose-limiting factor related to cisplatin is nephrotoxicity [3]. Acute kidney injury (AKI) is one of the major characteristics of cisplatin-induced renal toxicity, which is related to high morbidity and mortality [4]. Recently, several studies have reported that cisplatin-induced AKI can be detected in early phase using novel biomarkers [5,6,7,8]. In the case AKI appears and its major cause is not cured, it may be hard to restore renal function, and even more complications can follow [9].

AKI, characterized mainly by tubular cell necrosis, is a key indicator for assessing side effects in the examination of several therapeutic agents and other interventions [10]. Oxidative stress is an imbalance between the oxidation and reduction reactions, resulting in the production of reactive oxygen species (ROS). Previous studies have reported that cisplatin-induced AKI is related to exacerbation of antioxidant state, involving an elevation of malondialdehyde (MDA) content as the final product of lipid peroxidation [11] and reduced levels of antioxidant enzymes in renal tissue [12]. Several enzymes also contribute to the ROS generation in vivo. Among them, nicotinamide adenine dinucleotide phosphate oxidase (Nox) can be stimulated to generate ROS within a few minutes by various growth factors, such as cytokines and hormones [13]. NADPH oxidase type 4 (Nox4) is a member of the Nox family and highly expressed in the kidney, mostly in the highly metabolic proximal tubular compartment [14]. Additionally, ROS-mediated oxidative stress triggers the activation of series of signaling proteins pathway, such as inflammatory pathway nuclear factor-κB (NF-κB) [15]. ROS also plays a main role via inducing the activation of Caspases in cisplatin-induced tubular cell apoptosis. Caspases are a family of cell death proteases that play an essential role in the execution phase of apoptosis [16]. Generally, the mechanisms implicated in cisplatin-induced AKI seem to be pluralistic, including inflammation, oxidative stress and apoptosis [17]. Thus, an effective clinical agent is considered necessary for preventing the progression of AKI induced by cisplatin.

Several studies have focused currently on traditional herbal medicines to evaluate novel therapeutic drugs for the therapy of AKI. Various herbal medicines, including pomegranate (*Lythraceae*), *Prosthechea michuacana* (*Orchidaceae*), *Zingiber officinale* (*Zingiberaceae*) and Red ginseng (family *Araliaceae*) have been shown protective effects on cisplatin-induced AKI with vivo experiments [18,19,20]. *Panax quinquefolius* (PQ), namely American ginseng, is considered to be one of the most well-known perennial herbal plants of genus *Panax* (family *Araliaceae*) in the United States and Canada, and its roots and rhizomes have been used as drugs, dietary supplements, and foods for diabetes and cardiovascular diseases treatment for more than 300 years in China [21,22,23]. More pharmacological activities of PQ have been discussed in the previous study, involving anti-inflammatory [24], anti-oxidation [25], hypoglycemic effect [26], etc. The main active ingredient of PQ is ginsenosides, a diverse group of steroidal saponins [27], which is considered to be the predominant component of the leaves of *P. quinquefolius*. Recent investigation showed that the saponins from leaves of *P. quinquefolius* (PQS) have a variety of pharmacological activity and can be applied in clinic. Wang et al. reported that PQS had a beneficial effect on the treatment of coronary heart disease [28] and PQS is one of the most frequently therapies used in clinical practice for acute myocardial infarction [29]. Additionally, PQS attenuated oxidative stress injury by intermittent high glucose in cultured human umbilical vein endothelial cells [30] and the extracts from leaves of *P. quinquefolius* also have anti-inflammation, free radical scavenging and other pharmacological activities in arteriosclerosis [31]. We speculate that it is possible that treatment with PQS supplementation may ameliorate cisplatin-induced lipid peroxidation, inflammation, and reduced renal tubular necrosis in mice.

In this paper, the renoprotective effects of PQS against cisplatin-induced AKI surveyed on a mouse model was first proposed. In addition, the feasible molecular mechanisms underlying this nephroprotective effect are discussed, involving antioxidant, anti-inflammatory, and anti-apoptotic activity.

## 2. Results

### 2.1. Effects of P. quinquefolius (PQS) on Renal Dysfunction in Cisplatin-Treated Mice

**Figure 1:**
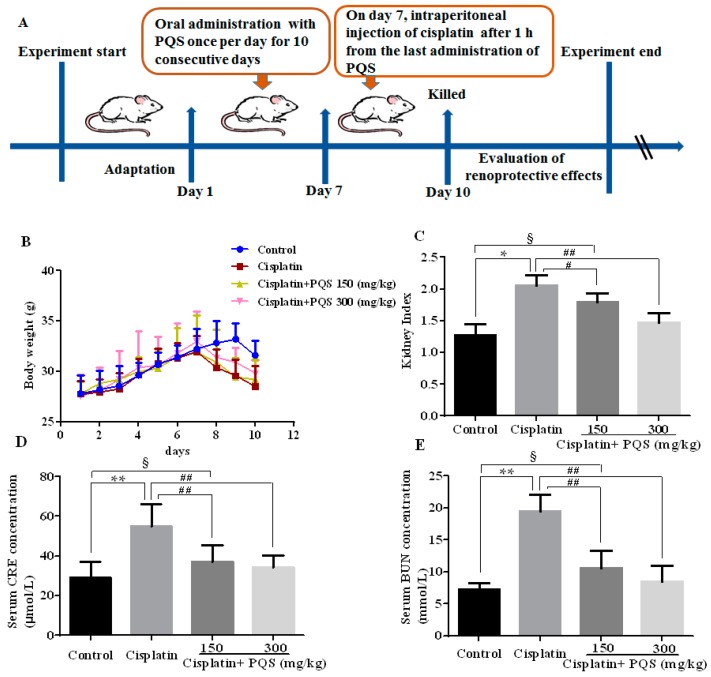
Experimental design of renoprotective effect of *P. quinquefolius* (PQS) on mice was summarized (**A**). Effects of PQS on: body weight change (**B**); kidney index (**C**); the level of serum creatinine (CRE) (**D**); and the level of blood urea nitrogen (BUN) (**E**) in cisplatin-induced acute kidney injury (AKI). All data are expressed as mean ± S.D., *n* = 8. ^§^ *p* < 0.05, * *p* < 0.05, ** *p* < 0.01 vs. control group; ^#^ *p* < 0.05, ^##^ *p* < 0.01 vs. cisplatin control group. Notes: standard deviation, S.D.; institute of cancer research, ICR.

Experimental design of renoprotective effect of PQS on mice was summarized in Figure 1A. Single treatment of cisplatin (20 mg/kg) caused noticeable weight loss and raised relative kidney index in the cisplatin control group compared with control group (*p* < 0.05). However, these changes in body weight and organ index were significantly dose-dependently attenuated by PQS at doses of 150 and 300 mg/kg, as shown in Figure 1B,C. Control group showed no significant difference compared with that in the mice treated with PQS high dose group (*p* < 0.05 or *p* < 0.01).

To further assess whether PQS preserved renal function, the serum levels of serum creatinine (CRE) and blood urea nitrogen (BUN) were determined in each group (Figure 1D,E). Similarly, the contents of CRE and BUN, a signal of kidney injury, were both uncommonly increase after cisplatin injection, indicating a serious injury to kidney tissues. PQS administration at the dosage of 150 and 300 mg/kg showed a dose-dependently protective effect, as illustrated by standardization of CRE and BUN as compared with cisplatin control group (*p* < 0.01).

### 2.2. Effects of PQS on Oxidative Stress of Kidney in Cisplatin-Treated Mice

**Figure 2:**
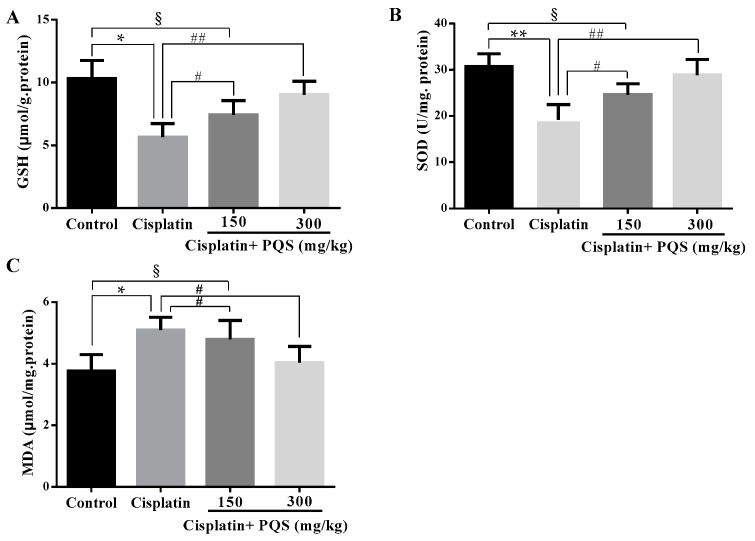
Effects of PQS on the levels of: glutathione (GSH) (**A**); superoxide dismutase (SOD) (**B**); and malondialdehyde (MDA) (**C**) in cisplatin-induced AKI. All data are expressed as mean ± S.D., *n* = 8. ^§^ *p* < 0.05, * *p* < 0.05, ** *p* < 0.01 vs. control group; ^#^ *p* < 0.05, ^##^ *p* < 0.01 vs. cisplatin group.

**Figure 3:**
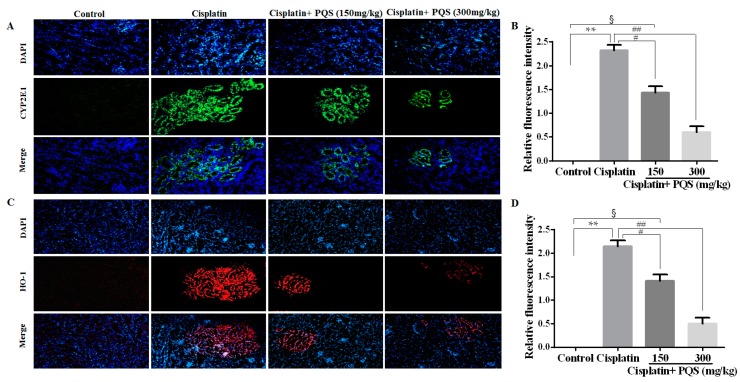
Effects of PQS on the expression of: enzyme cytochrome P450 E1 (CYP2E1) (**A**); and heme oxygenase-1 (HO-1) (**C**). The expression level of CYP2E1 (Green) and HO-1 (Red) in tissue section isolated from different groups was assessed by immunofluorescence. Column chart (**C**,**D**) shows relative fluorescence intensity. Representative immunofluorescence images were taken at 400×. 4′,6-Diamidino-2-phenylindole (DAPI) (Blue) was used as a nuclear counterstain. ^§^ *p* < 0.05, ** *p* < 0.01 vs. control group; ^#^ *p* < 0.05, ^##^ *p* < 0.01 vs. cisplatin group.

**Figure 4:**
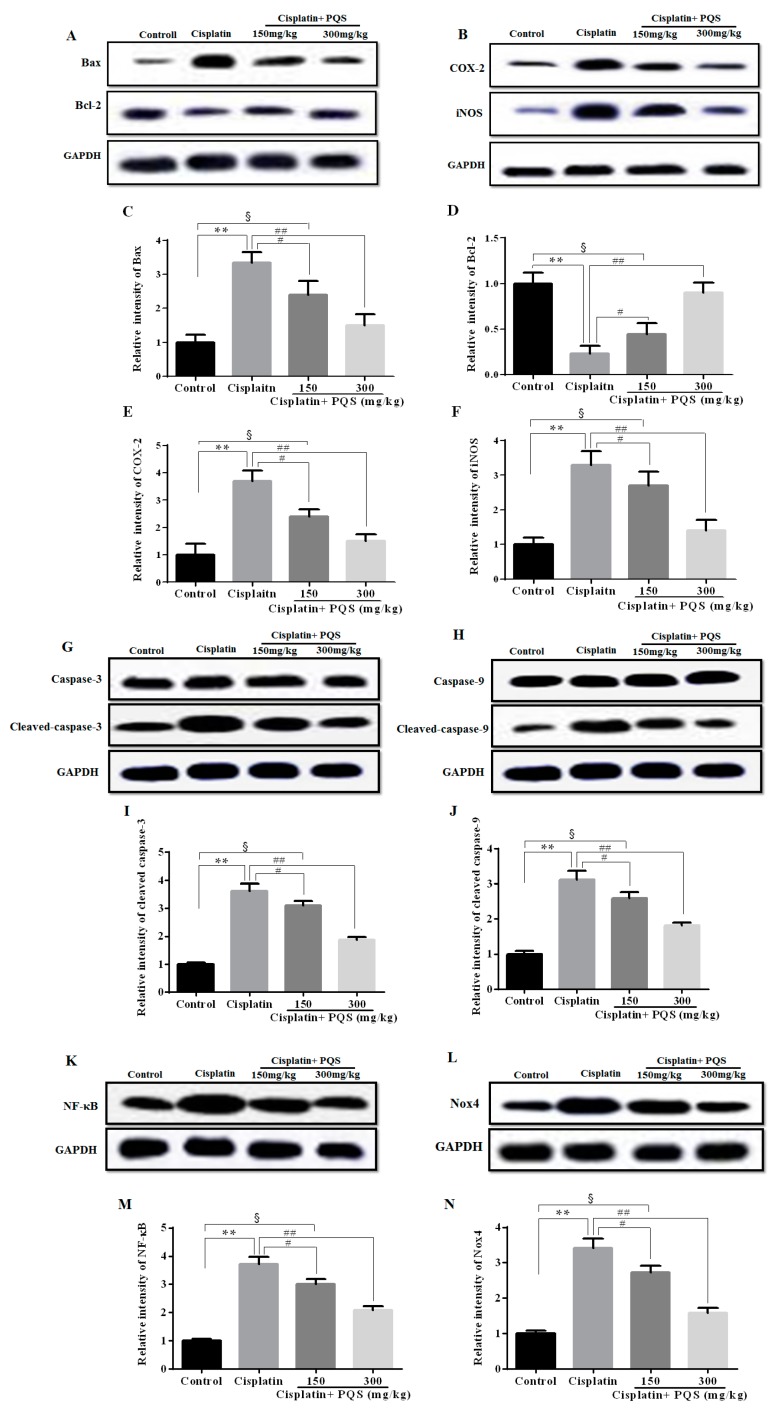
Effects of PQS on the protein expression of: Bax, Bcl-2 (**A**); COX-2, iNOS (**B**); cleaved Caspase-3 (**G**); cleaved Caspase-9 (**H**); NF-κB (**K**); and Nox4 (**L**). Column chart show antibodies relative intensity of: Bax (**C**); Bcl-2 (**D**); COX-2 (**E**); iNOS (**F**); cleaved Caspase-3 (**I**); cleaved Caspase-9 (**J**); NF-κB (**M**); and Nox4 (**N**). The protein expression was examined by Western blotting analysis in kidney tissues from control, cisplatin, cisplatin + PQS (150 mg/kg), and cisplatin + PQS (300 mg/kg). ^§^ *p* < 0.05, ** *p* < 0.01 vs. control group; ^#^ *p* < 0.05, ^##^ *p* < 0.01 vs. cisplatin control group. Notes: glyceraldehyde-3-phosphate dehydrogenase, NAPDH; inhibitor of NF-κB, IκB; horseradish peroxidase, HRP.

As previously described, oxidative stress injury participated in the mechanisms of cisplatin-induced AKI [32]. As indicated in Figure 2, cisplatin treatment caused conspicuous reduction of glutathione (GSH) level and superoxide dismutase (SOD) activity accompanied by increase of MDA content, compared with control group (*p* < 0.05 or *p* < 0.01). However, administration with 150 and 300 mg/kg of PQS reduced MDA content and restored antioxidant capacity, as illustrated via the increase of GSH level and SOD activity (*p* < 0.05 or *p* < 0.01). These data suggested that PQS mitigated oxidative damage in kidney tissues by up-regulating anti-oxidant enzyme activity. To confirm whether oxidative stress is interrelated to the evolvement of cisplatin-induced AKI in vivo, the expression levels of NADPH oxidase enzyme Nox4, drug-metabolizing enzyme cytochrome P450 E1 (CYP2E1) and cytoprotective enzyme heme oxygenase-1 (HO-1) were examined. The result showed the expression of CYP2E1 and HO-1 were low in control and PQS-treated mice (Figure 3A,C) and apparently elevated following cisplatin challenge. Meanwhile, the results from Western blotting showed that the expression level of Nox4 increased after cisplatin exposure was decreased by PQS (Figure 4L). These consequences suggested that PQS supplementation protected the kidneys against cisplatin-induced oxidative stress.

### 2.3. Effects of PQS on Histopathological Changes of Kidney in Cisplatin-Treated Mice

**Figure 5:**
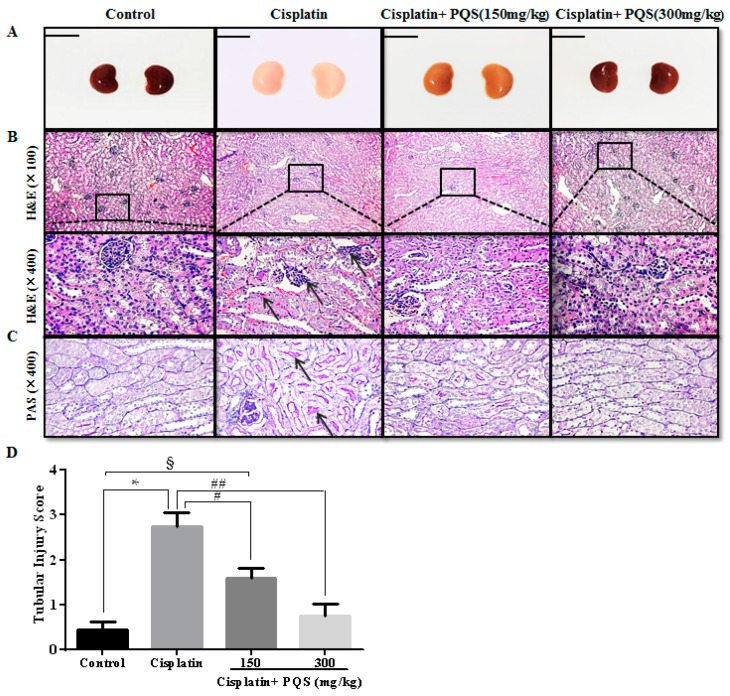
Morphological changes of the kidneys (**A**); histological examination of morphological changes in kidney tissues, renal tissues stained with H&E (100× and 400×) (**B**); arrows show necrotic cell and inflammatory infiltrate cells (PAS (400×)) and arrows show glycogen accumulation in renal tubules (**C**); and column chart shows renal tubular injury score (**D**). All data are expressed as mean ± S.D., *n* = 8. ^§^ *p* < 0.05, * *p* < 0.05 vs. control group; ^#^ *p* < 0.05, ^##^ *p* < 0.01 vs. cisplatin group. Notes: periodic acid-schiff stain, PAS.

The morphological changes in the kidneys are shown in the Figure 5A. The kidney tissues in cisplatin mice clearly revealed tubular necrosis and inflammatory infiltration (Figure 5B). However, in the group treated with high dose of PQS (300 mg/kg), tubules distinctly noticed histologically regular and no inflammatory infiltrate cells and necrosis were noticed in kidney tissues. In addition, we found that, in the PAS staining (Figure 5C), the renal tubules had fewer glycogen deposits, with lighter color, and no pathological changes were found in the control group. A great number of glycogens were deposited in the renal tubules and hypochromatosis in the cisplatin control group. Nevertheless, pretreatment with PQS low dose group evidently reduced tubular cells necrosis and glycogen deposition, PQS high dose group of cell morphology basically returned to control.

### 2.4. Effects of PQS on Inflammation of Kidney in Cisplatin-Treated Mice

**Figure 6:**
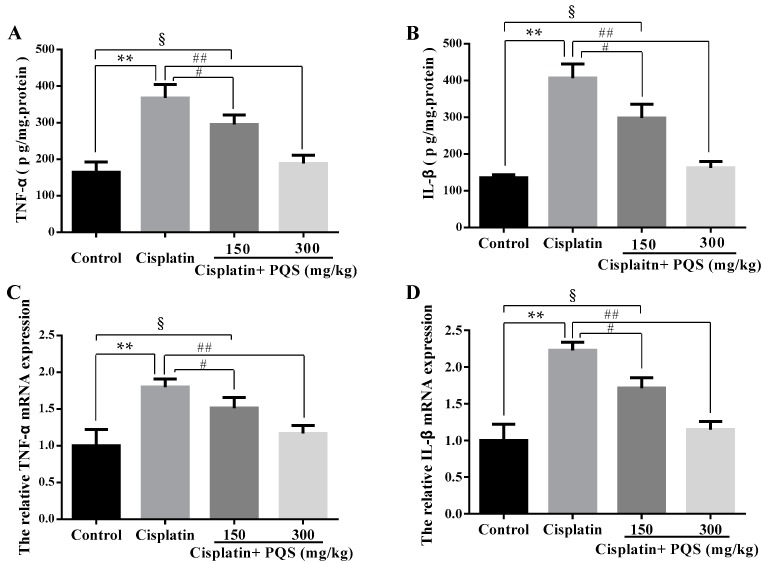
Effects of PQS on the levels of: tumor necrosis factor-α (TNF-α) (**A**); and interleukin-1β (IL-1β) (**B**) in cisplatin-induced AKI. The relative expressions: *TNF-α* (**C**); and *IL-1β* (**D**) in mice were evaluated by quantitative real-time PCR (qRT-PCR). All data are expressed as mean ± S.D., *n* = 8. ^§^ *p* < 0.05, ** *p* < 0.01 vs. control group; ^#^ *p* < 0.05, ^##^ *p* < 0.01 vs. cisplatin control group. Notes: enzyme linked immunosorbent assay, ELISA; optical density, OD.

**Figure 7:**
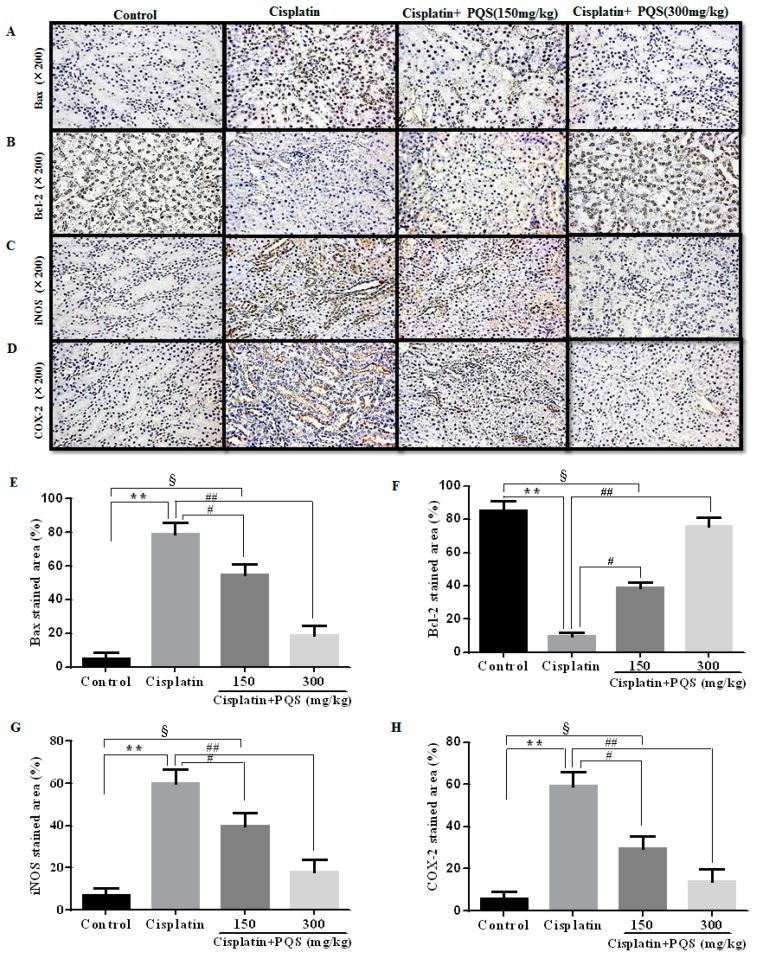
Effects of PQS on the expression of: Bax (**A**); Bcl-2 (**B**); inducible nitric oxide synthase (iNOS) (**C**); and cyclooxygenase-2 (COX-2) (**D**). Column chart show antibodies stained area of: Bax (**E**); Bcl-2 (**F**); iNOS (**G**); and COX-2 (**H**). The protein expression was examined by immunohistochemistry in kidney tissues from control, cisplatin, cisplatin + PQS (150 mg/kg), and cisplatin + PQS (300 mg/kg). ^§^ *p* < 0.05, ** *p* < 0.01 vs. control group; ^#^ *p* < 0.05, ^##^ *p* < 0.01 vs. cisplatin control group. Notes: b-cell lymphoma-2 protein, Bcl-2; Bcl-2-associated x protein, BAX; bovine serum albumin, BSA.

The previous study has confirmed that oxidative stress is related with release of pro-inflammatory cytokines, including tumor necrosis factor-α (TNF-α) and interleukin-1β (IL-1β) in cisplatin-induced AKI [33]. As indicated in Figure 6A,B, single cisplatin injection resulted in obviously higher levels of TNF-α and IL-1β in serum as compared with the control group. However, these increases were reversed after PQS administration in a dose-independent manner (*p* < 0.01, *p* < 0.05). Besides, to further assess the effects of PQS against cisplatin-induced inflammatory response in the kidney tissues, *TNF-α* and *IL-1β* mRNA expression levels were estimated by quantitative real-time PCR (qRT-PCR) (Figure 6C,D). PQS attenuated the increase pro-inflammatory cytokine expression levels following cisplatin exposure (*p* < 0.05 or *p* < 0.01). Concomitantly, to further investigate the mechanisms underlying the beneficial effects of PQS on cisplatin induced renal inflammation, the levels of NF-κB, cyclooxygenase-2 (COX-2) and inducible nitric oxide synthase (iNOS) were determined in all groups (Figure 4B,K and Figure 7C,D). Positive expression area of these protein levels of cisplatin control group was obviously elevated compared with control group, whereas PQS treatment for 10 days led to dose-dependent reduction. These protein expression levels in the control mice showed no significant difference compared with PQS group (300 mg/kg) (*p* < 0.05 or *p* < 0.01).

### 2.5. Effects of PQS on Apoptosis of Kidney in Cisplatin-Treated Mice

To measuring the degree of apoptosis in kidneys, the protein expressions of the cleaved Caspase-3, cleaved Caspase-9, anti-apoptotic factor Bcl-2 and the pro-apoptotic factor Bax were determined in each experimental group. As shown in Figure 7A,B. The rates of positive expressions of Bax located in the cell nucleus were observed to be noticeably lower in PQS administration group (300 mg/kg) different from cisplatin control group. The positive expression of Bcl-2 was similar with Bax and it has a significant elevation in the PQS group with 300 mg/kg compared to cisplatin group (*p* < 0.01). The results of Western blotting showed that cisplatin exposure significantly increased the expression levels of Bax, cleaved Caspase-3 and Caspase-9, and decreased the expression level of Bcl-2 when compared with the control group. However, these changes could be effectively reversed with PQS pretreatment (Figure 4A,G,H) (*p* < 0.05 or *p* < 0.01).

**Figure 8:**
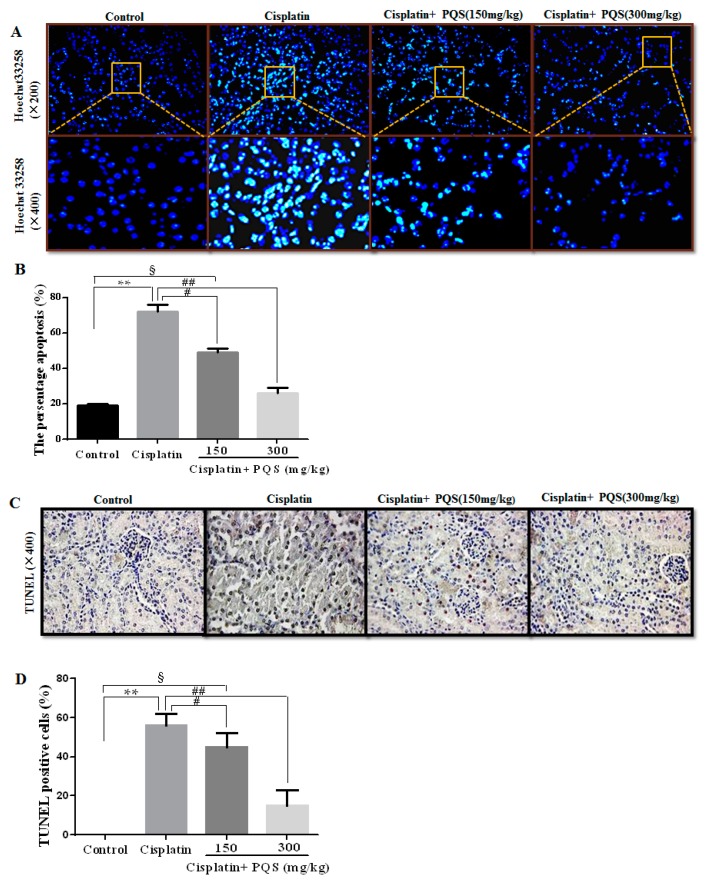
Histological examination of morphological changes in kidney tissues, renal tissues stained with: Hoechst 33258 (200× and 400×) (**A**); column chart showing renal tubular cell apoptosis (**B**); TUNEL staining (400×) (**C**); and the presence of TUNEL positive cells measured by the image analyzer (**D**). All data are expressed as mean ± S.D., *n* = 8. ^§^ *p* < 0.05, ** *p* < 0.01 vs. control group; ^#^ *p* < 0.05, ^##^ *p* < 0.01 vs. cisplatin group. Notes: terminal deoxynucleotidyl transferase-mediated dUTP-biotin nick end labeling assay, TUNEL.

Aimed to define whether PQS administration decreased renal tubular cell apoptosis against cisplatin-induced AKI, Hoechst 33258 staining was employed to determine the apoptosis extent in renal tubular cells. The results revealed that the nuclear fragmentations and condensations in cisplatin group were uncommonly higher when compared with control group (Figure 8A). Nevertheless, after PQS administration, a larger number of cell nucleus appeared round-shaped nuclei with homogeneous fluorescence intensity and regular contours compared with cisplatin control group (*p* < 0.05 or *p* < 0.01). Moreover, in the recent studies, the apoptosis in the kidney tissues were evaluated and quantified by the TUNEL assay staining, which revealed renal tubular epithelial cell apoptosis after cisplatin exposure. Interestingly, PQS pretreatment at low dose obviously reduced the number of cisplatin-induced TUNEL-positive cells, and more significant decrease almost similar to the control group at high dose (Figure 8C). These results showed that PQS exerted suppressive effects against cisplatin-induced renal proximal tubule apoptosis.

## 3. Discussion

In the present study, PQS was endowed with significantly nephroprotective properties against cisplatin-induced nephrotoxicity. Though our work was similar to a previous report showing that ginsenoside Rg5 ameliorated acute kidney injury with the same mouse model via inhibiting oxidative stress, inflammation and apoptosis [17], the in-depth molecular mechanisms of cisplatin-induced renal toxicity by PQS were explored in our study. It needs to be emphasized that the process of isolation and purification of ginsenoside Rg5, as an individual rare ginsenoside, was certainly difficult and time consuming. It is very meaningful for us to seek an alternative extract that has the similar or better effect in cisplatin-induced nephrotoxicity. As non-traditional medicinal part of *P. quinquefolium*, the total saponins from its leaves are relatively easy and cheap to extract. Moreover, in the current work, PQS were explored in depth for its significant renoprotective effect. The present findings also enhanced its medicinal value and opened a new market for leaves of *P. quinquefolium* to alleviate the patient’s kidney injury in clinical research.

Cisplatin has been studied for more than 40 years and has made a significant contribution to aggressive cancer, but, in more than 10% of patients, conventional cisplatin note can cause acute renal toxicity and even cause irreversible damage. In the current work, we demonstrated that PQS pretreatment significantly attenuated cisplatin-induced AKI through the decrease of oxidative stress, inflammation response and apoptosis. Our present work indicated that cisplatin treatment caused typical clinical symptoms and pathological changes in cisplatin-treated mice, including body weight loss, relative kidney index increase, inflammatory infiltration, and necrosis. In addition, cisplatin exposure induces damage to renal vasculature resulting declined in glomerular filtration rate [1]. This caused renal dysfunction, urging acute renal failure and the elevation of serum BUN and CRE levels. PQS significantly inhibited the elevation of the kidney index, BUN and CRE levels and weight loss by cisplatin, suggesting positive effects of PQS on renal dysfunction in cisplatin-treated mice.

Several studies have revealed that single cisplatin exposure generated overproduction of free radicals, and then caused oxidative stress damage and lipid peroxidation in kidney tissues [34,35,36]. Previous reports have also clarified that oxidative stress is a central pathogenic factor in cisplatin-induced AKI, which showed that cisplatin undermined anti-oxidant defense mechanisms in the kidneys followed by a significant decrease in the contents of SOD and GSH [37]. Concomitantly, cisplatin exposure increased the kidney content of MDA, which a major marker of lipid peroxidation. In the present investigation, pretreatment with PQS for 10 days obviously restrained the increase of MDA and decline of GSH and SOD in a dose-dependent manner. Nox is an enzyme that produces free radicals as its main product [38]. Seven members are included in the Nox family: Nox1 to Nox5 and two dual oxidases. Among them, Nox4 has been considered as a major source of ROS in renal cells, such as endothelial cells, vascular smooth muscle cells and epithelial cells of various tubules of the kidney [39], which also plays important roles in renal oxidative stress and kidney injury [40]. The research showed that protein expression level of Nox4 increased after cisplatin treatment, whereas was markedly decreased by PQS in our work. Generally, CYP2E1 mediated biotransformation of cisplatin emerges ROS, involving hydrogen peroxide and hydroxyl radical [41], well known inducers of lipid peroxidation [42]. Importantly, the finding in our work was consistent with a previous study which has confirmed a leading role of drug-metabolizing enzyme CYP2E1 against cisplatin-induced renal injury [43]. Simultaneously, the increase in CYP2E1 level was accompanied by the overexpression of HO-1 cytoprotective enzyme, showing mobilization of the antioxidant defense system [44]. Immunofluorescence results showed that the expression of CYP2E1 and HO-1 increased after cisplatin treatment was reduced by PQS in present work. As described above, our findings illustrated which PQS pretreatment recovered antioxidant ability by suppressing oxidative stress.

Numerous studies demonstrated that pro-inflammatory cytokines possess an indispensable role in the pathogenesis against cisplatin-induced renal toxicity [45]. NF-κB is a pro-inflammatory transcription factor of inflammatory factors that is sequestered in the cytoplasm via binding to the inhibitory IκB protein. Once stimulated by viral, bacterial and other pathogens, IκB is degraded by the proteasome, which frees NF-κB to translocate from the cytoplasm to the nucleus where it promotes the transcription of target genes, such as *TNF-α* and *IL-1β* [46]. Moreover, several studies have demonstrated the significant part of TNF-α and IL-1β in the pathogenesis against cisplatin renal injury [47]. Simultaneously, the up-regulation of iNOS and other inducible genes, such as COX-2 via the NF-κB pathway, is another central mechanism in inflammatory response processes [48]. The induction of COX-2, an inducible form of COX, may arise in tissue injury and it has been certified that COX-2 plays a major role against cisplatin-induced AKI. Accumulating evidence showed that COX-2 and iNOS are obviously expressed at positions of inflammation. Moreover, iNOS acts in synergy with COX-2 to accelerate the inflammatory response [49,50]. In this study, to explore the potential molecular mechanisms the renoprotective effects of PQS against cisplatin-induced inflammation in depth, we measured the expression levels in NF-κB, COX-2 and iNOS. Western blotting and immunohistochemistry analysis of the results revealed that PQS pretreatment significantly down-regulated the increase in expression levels of NF-κB, COX-2 and iNOS after cisplatin exposure. Moreover, qRT-PCR results revealed that cisplatin administration elevated the expression levels in *TNF-α* and *IL-1β*. Nevertheless, the increase of inflammatory cytokines levels against cisplatin-induced mice was dramatically suppressed by PQS. The possible molecular mechanisms involved in suppression of such cytokines by PQS. In short, the results suggested that PQS could be served as an anti-inflammatory agent against cisplatin-induced AKI.

There is growing evidence that cisplatin-induced AKI is related to apoptosis [51]. Apoptosis acts as a key role of cell death against cisplatin-induced renal toxicity, and then multitudinous investigations have indicated renal tubular cell apoptosis following cisplatin management [52]. There are two of the considerable members regarding renal tubular cell apoptosis in Bcl-2 family containing the pro-apoptotic protein Bax and the anti-apoptotic protein Bcl-2 [53]. Another important mechanism underlying the AKI of cisplatin is the pro-apoptotic effects caused by inducing the activation of Caspases [54]. Previous studies have also reported association between Caspases family members and cisplatin-induced apoptosis. Cisplatin may cause mitochondrial release of cytochrome c and Caspase-9 and Caspase-3 activation [55]. For evaluated agree of apoptosis in renal tissues following single cisplatin injection, the effects of PQS were assessed by cleaved Caspase-3, cleaved Caspase-9, pro-apoptotic factor Bax and anti-apoptotic factor Bcl-2 in every pretreatment group. The findings from protein detection analyses of kidneys markedly illustrated that the expression levels of Bax, cleaved Caspase-3 and cleaved Caspase-9 were suppressed, while the expression level of Bcl-2 was relatively activated. Additionally, the results of TUNEL staining and Hoechst 33258 staining displayed which the apoptosis rate in renal tissues were clearly reduced by PQS when compared with cisplatin control group. In summary, the above results demonstrated that PQS extenuated apoptosis of kidney tissues and expressed renoprotective effects on AKI in cisplatin-induced mice.

## 4. Materials and Methods

### 4.1. Materials and Kits

PQS extracted from the leaves of *P. quinquefolium* (American ginseng) was prepared and quantified in our laboratory as described previously [56]. Briefly, PQS was refluxed with 70% ethanol for three times and then separated by column chromatography with AB-8 resin. PQS analysis was built on a Hypersil ODS column and detected by high performance liquid chromatography with UV detection (HPLC-UV) at 203 nm. Seven ginsenosides (Rg1, Re, Rb1, Rc, Rb2, Rb3, and Rd) in PQS were determined by comparing their retention times with corresponding standard compounds. The final product consisted of 11.94% Rg1, 3% Re, 2.62% Rb1, 4.01% Rc, 10.04% Rb2, 3.70% Rb3, and 27.49% Rd, accounting for 62.8% of the PQS, and other minor ginsenosides.

Cisplatin, with purity more than 99%, was supplied from Sigma Chemicals (St. Louis, MO, USA). Hematoxylin and Eosin (H&E), periodicacid–Schiff (PAS) dye kits, malondialdehyde (MDA), glutathione (GSH), superoxide dismutase (SOD), blood urea nitrogen (BUN) and creatinine (CRE) commercial assay kits were obtained from Nanjing Jiancheng Bioengineering Research Institute (Nanjing, China). Rabbit monoclonal of Caspase-3, cleaved Caspase-3, Caspase-9, cleaved Caspase-9, Bax, Bcl-2, NF-κB, COX-2, iNOS, GAPDH, cytochrome P450 E1 (CYP2E1), heme oxygenase-1 (HO-1) and secondary antibodies were purchased from Cell Signaling Technology (Danvers, MA, USA) or DBOSTER Bio-Engineer Co., Ltd. (Wuhan, China). Nox4 antibody was obtained Proteintech Co., Ltd. (Wuhan, China). RNAiso Plus, PrimeScript^®^RT reagent Kit (Perfect Real Time) and SYBR^®^Premix Ex TaqTM II (Tli RNaseH Plus) were received from TaKaRa Biotechnology Co., Ltd. (Dalian, China). TUNEL apoptosis detection kits were provided with Roche Applied Science (No. 11684817910). Hoechst 33258 dye kits were obtained from Shanghai Beyotime Co., Ltd. (Shanghai, China). DyLight 488-labeled and SABC-Cy3 secondary antibodies were provided by BOSTER Bio-Engineer Co., Ltd. (Wuhan, China). The ELISA kits of mouse TNF-α and IL-1β were purchased from R&D systems (Minneapolis, MN, USA). All other chemicals were of the highest grade commercially available.

### 4.2. Animals and Experimental Protocol

Six-week-old male ICR mice, weighting 22–25 g, were provided by YISI Experimental Animals Co., Ltd. (Changchun, China). The mice were given a standard laboratory diet and water ad libitum and maintained at 12 h light/dark cycle at constant temperature (25 ± 2 °C) and humidity (60 ± 10%). All animals handing procedures were performed in strict accordance with the Guide for the Care and Use of Laboratory Animals (Ministry of Science and Technology of China, 2006). All the animal tests were carried out consistent with experimental practices and standards, which were authorized by Ethical Committee of Jilin Agricultural University (2016-01-Permit Number: ECLA-JLAU 2016-016).

After acclimation for one week, the mice were randomly divided into for experimental groups (8 mice per group), including a control group, cisplatin group (25 mg/kg), cisplatin + PQS groups (150 and 300 mg/kg). PQS was suspended in 0.05% carboxymethylcellulose sodium (CMC-Na) before used. Mice were administered intragastrically with PQS at the dose of 150 and 300 mg/kg daily for ten continuous days. The mice in cisplatin group and PQS administration groups received a single intraperitoneal injection of cisplatin with 20 mg/kg on the 7th day after 1 h from the last administration of PQS to induce AKI in mice.

Mice were sacrificed at 72 h following cisplatin injection under general anesthesia. Weighting, and blood and organ samples collection were performed immediately. The right kidney was promptly frozen in liquid nitrogen and stored at −80 °C till analysis and the left kidney was fixed in 10% neutral buffered formalin. Subsequently, serum samples were segregated using refrigerated centrifuge at 3000× *g* and stored at −20 °C used for subsequent analysis. The kidneys were gathered and weighted, kidney index (mg/g) kidney weight/body weight was calculated as the kidney index.

### 4.3. Assessment of Biochemical Parameters

The serum levels of BUN and CRE were measured with commercially diagnostic kits (Nanjing Jiancheng Bioengineering Research Institute, Nanjing, China) judging by manufacturer’s protocols.

The levels of GSH and SOD were examined with commercially available assay kits as described previously. In brief, the homogenates of thawed and homogenized were filtered and centrifuged with refrigerated centrifuge at 4 °C. Subsequently, half homogenates were accustomed to evaluate lipid peroxidation via determining thiobarbituric acid reactive substances (TBARS) and was expressed of the malondialdehyde (MDA) content. Then, the protein concentrations were measured by Bradford protein assay with bovine serum albumin as the standard (Beyotime Biotechnology, Shanghai, China). The enzyme activity was expressed as unit per milligram of protein.

### 4.4. Determination of Inflammatory Cytokines

The serum levels of TNF-α, IL-1β were examined with a commercially available ELISA kits (R&D Systems) as described previously. In brief, TNF-α and IL-1β were added to 96-well plate containing antibody and combined with HRP-labeled antibody (TNF-α, IL-1β) to form complex (an antibody-antigenic-antibodies labeled with enzyme compound), finally, the substrate TMB was added for measuring the OD value within 15 min.

### 4.5. Histopathological Examination

Histopathological examination was executed as mentioned earlier [57]. The left renal tissues were fixed in 4% neutral formaldehyde solution. Further, the tissues were dehydrated, embedded in paraffin then cut into 5 μm-thickness slice. Then, all slices were stained with H&E staining solution and PAS reagents for histopathological analysis and further detected by light microscopy (Leica DM750, Solms, Germany).

Kidney slices were measured and coded with two independent trained observers. PAS-stained sections of tubular injury were detected with a microscope and scored following the percentage of cortical tubules having epithelial necrosis: 0%, control; 1, <10%; 2, 10–25%; 3, 26–75%; or 4, >75% [58].

### 4.6. Hoechst 33258 Staining Analysis

Hoechst 33258 staining analysis was executed as mentioned earlier with minor modifications [59]. Firstly, the above treated 5 μm-thickness kidney slices were stained by Hoechst 33258 with concentration of 10 μg/mL. Next, the sections were rinsed 3 times in PBS for 10 min and stained nuclei were visible below UV excitation. Finally, the slides were photographed with a fluorescent microscope (Leica TCS SP8, Solms, Germany).

### 4.7. TUNEL Staining Analysis

To measure the extent of apoptosis in kidney after cisplatin exposure, the TUNEL analysis was determined. TUNEL evaluation was carried out as mentioned earlier with minor modification [17]. Typically, employing in situ apoptosis detection kit (Mannheim, Germany) to discover apoptotic cells in the kidney tissues judging by the manufacturer’s instructions. Firstly, the kidney sections (5 μh thick) were installed on the slides, permeabilized by incubating with 100 μL of 20 µg/mL proteinase K solution for 15 min. Next, the sections were incubated with 100 μL of 0.3% H_2_O_2_ for 5 min and incubated by equilibration buffer and terminal deoxynucleotidyl transferase to inactivate endogenous peroxidase. Then, anti-digoxigenin-peroxidase conjugate was employed to incubate the sections. Finally, the utilization of diaminobenzidine demonstrated peroxidase activity in all tissue sections and the slices were counterstained with hematoxylin. TUNEL-positive cells were visualized with a Leica microscope (Leica TCS SP8, Solms, Germany).

### 4.8. Immunohistochemistry (IHC) and Immunofluorescence Analysis

IHC analysis was carried out as mentioned earlier [60]. Firstly, aqueous alcohol solutions were used to deparaffinizing and rehydrating kidney sections (5 μm). The slices were blocked with endogenous peroxidases for 30 min at 37 °C, followed by rinsing 3 times in Tris-buffered saline (TBS 0.01 M, pH 7.4) for 10 min. Then, the slices were then irradiated in sodium citrate buffer (0.01 M, pH 6.0) in a microwave oven (medium high temperature) for 20 min. After standing to room temperature, slides were incubated with 1% BSA for 1 h and incubated overnight at 4 °C with initial antibodies involving Bax (1:200), Bcl-2 (1:200), iNOS (1:200) and COX-2 (1:200) in a humid environment. The sections were rinsed in TBS and then incubated in HRP-conjugated secondary anti-bodies for 30 min at 37 °C. Next, slices were rinsed in TBS and incubated with labeled streptavidin-biotin for 30 min. After rinsing in TBS, the sections were visualized with DAB and counterstained with hematoxyline followed. Finally, the sections were washed in running water for 5 min, dehydrated with an alcohol series and cleared with xylene. Immunostaining intensity was analyzed by light microscopy (Leica DM750, Solms, Germany).

The immunofluorescence staining of kidney tissues was guided as described [61], which was executed on tissue sections treated as depicted for IHC analysis. Briefly, the sections were incubated with the primary antibodies against rabbit anti-mouse CYP2E1 (1:200) and heme oxygenase-1 (HO-1) (1:200) at 4 °C overnight. The slides were covered with DyLight 488-labeled and SABC-Cy3 secondary antibodies (BOSTER, Wuhan, China) on the second day. Then, 4-6-diamidino-2-phenylindole (DAPI) was used for nuclear staining and immunofluorescence staining was visible with a Leica microscope (Leica TCS SP8, Solms, Germany).

### 4.9. Real-Time PCR Analysis

Total RNA in kidney tissues was isolated with RNAiso Plus, and RNA was reverse transcribed to cDNA by PrimeScriptTM RT Reagent Kits (Perfect Real Time) judging by manufacturer’s instructions. The qRT-PCR was performed on 7500 real-time PCR System (Applied Biosystems, Foster City, CA, USA). The specific primers acquired from TaKaRa Biotechnology Co., Ltd. (Dalian, China) used for enlarging the specific genes were depict in Table 1. The gene expression was controlled to GAPDH and estimated by 2(−ΔΔ*C*t) method.

### 4.10. Western Blotting Analysis

Western blotting analysis was executed as mentioned earlier [62]. Firstly, the kidney tissues were cracked by using Radio Immunoprecipitation Assay (RIPA) buffer. The proteins (50 µg/lane) were separated with 12% SDS polyacrylamide gels and transferred to a polyvinylidene difluoride (PVDF) membrane. Then, the membrane was closed using 5% non-fat milk in Tris-buffered saline (TBS) containing 0.1% Tween-20 for 2 h in a room temperature situation, after incubated with primary antibodies at 4 °C overnight. Next, the membrane was washed three times from TBST for 5 min each time and incubated for 1 h at a room temperature environment using secondary antibodies later. Finally, the singles were inspected by Emitter Coupled Logic (ECL) substrate (Pierce Chemical Co., Rockford, IL, USA). The intensity of the bands were analyzed with computer Image plus 6.0 software (Media Cybernetics, Rockville, MD, USA).

### 4.11. Statistical Analysis

Results were expressed as means ± standard deviation (SD) of eight animals in each group and were analyzed with SPSS 17.0 (SPSS, Chicago, IL, USA). The Data compared by one-way analysis of variance (ANOVA) with post hoc tests to cisplatin administration. *p* < 0.05 or 0.01 was considered significant.

## 5. Conclusions

In conclusion, the present findings revealed the protective effects of PQS against cisplatin-induced renal toxicity, and its mechanism may be due to the inhibition of oxidative stress, inflammation and apoptosis. Nevertheless, clinical trials are indispensable to confirm the renoprotective effect of PQS before its act as an agent for the administration against cisplatin-induced acute AKI.

## Figures and Tables

**Table 1:** Primers used in this study.

Primer Name	Nucleotide Sequence (5′–3′)
TNF-α forward	CTTCTCATTCCTGCTTGTG
TNF-α reverse	ACTTGGTGGTTTGCTACG
IL-1β forward	TTGTGGCTGTGGAGAAG
IL-1β reverse	CATCAGAGGCAAGGAGG
GAPDH forward	AGGTCGGTGTGAACGGATTTG
GAPDH reverse	GGGGTCGTTGATGGCAACA

## Abbreviations

PQ	*Panax quinquefolius*
AKI	Acute kidney injury
ROS	Reactive oxygen species
GSH	Glutathione
SOD	Superoxide dismutase
MDA	Malondialdehyde
BUN	Blood urea nitrogen
TNF-α	Tumor necrosis factor-α
CRE	Creatinine
IL-1β	Interleukin-1β
COX-2	Cyclooxygenase-2
iNOS	Inducible nitric oxide synthase
CYP2E1	Cytochrome P450 E1
HO-1	Heme oxygenase-1
NF-κB	Nuclear factor-κB
qRT-PCR	quantitative real-time PCR
Nox4	NADPH oxidase type 4
